# Single Nucleotide Polymorphisms of *NUCB2* and Their Genetic Associations with Milk Production Traits in Dairy Cows

**DOI:** 10.3390/genes10060449

**Published:** 2019-06-13

**Authors:** Bo Han, Yuwei Yuan, Yanhua Li, Lin Liu, Dongxiao Sun

**Affiliations:** 1 Department of Animal Genetics, Breeding and Reproduction, College of Animal Science and Technology, Key Laboratory of Animal Genetics, Breeding and Reproduction of Ministry of Agriculture and Rural Affairs, National Engineering Laboratory for Animal Breeding, China Agricultural University, Beijing 100193, China; bohan@cau.edu.cn (B.H.); workyyw0422@163.com (Y.Y.); 18800051836@163.com (Y.L.); 2 Beijing Key Laboratory of Dairy Cattle Genetic, Breeding and Reproduction, Beijing Dairy Cattle Center, Beijing 100192, China; liulin@bdcc.com.cn

**Keywords:** dairy cattle, milk yield and composition, SNP, genetic effect, TFBS

## Abstract

We previously used the RNA sequencing technique to detect the hepatic transcriptome of Chinese Holstein cows among the dry period, early lactation, and peak of lactation, and implied that the nucleobindin 2 (*NUCB2*) gene might be associated with milk production traits due to its expression being significantly increased in early lactation or peak of lactation as compared to dry period (*q* value < 0.05). Hence, in this study, we detected the single nucleotide polymorphisms (SNPs) of *NUCB2* and analyzed their genetic associations with milk yield, fat yield, fat percentage, protein yield, and protein percentage. We re-sequenced the entire coding and 2000 bp of 5′ and 3′ flanking regions of *NUCB2* by pooled sequencing, and identified ten SNPs, including one in 5′ flanking region, two in 3′ untranslated region (UTR), and seven in 3′ flanking region. The single-SNP association analysis results showed that the ten SNPs were significantly associated with milk yield, fat yield, fat percentage, protein yield, or protein percentage in the first or second lactation (*p* values <= 1 × 10^−4^ and 0.05). In addition, we estimated the linkage disequilibrium (LD) of the ten SNPs by Haploview 4.2, and found that the SNPs were highly linked in one haplotype block (D′ = 0.98–1.00), and the block was also significantly associated with at least one milk traits in the two lactations (*p* values: 0.0002–0.047). Further, we predicted the changes of transcription factor binding sites (TFBSs) that are caused by the SNPs in the 5′ flanking region of *NUCB2*, and considered that g.35735477C>T might affect the expression of *NUCB2* by changing the TFBSs for ETS transcription factor 3 (ELF3), caudal type homeobox 2 (CDX2), mammalian C-type LTR TATA box (VTATA), nuclear factor of activated T-cells (NFAT), and v-ets erythroblastosis virus E26 oncogene homolog (ERG) (matrix similarity threshold, MST > 0.85). However, the further study should be performed to verify the regulatory mechanisms of *NUCB2* and its polymorphisms on milk traits. Our findings first revealed the genetic effects of *NUCB2* on the milk traits in dairy cows, and suggested that the significant SNPs could be used in genomic selection to improve the accuracy of selection for dairy cattle breeding.

## 1. Introduction

Milk provides rich nutrients, protein, fatty acids, and vitamins, etc., for human [1], so the milk production traits are considered as the most important economic traits in the dairy industry, including milk yield, fat yield, fat percentage, protein yield, and protein percentage [2]. Researchers have attempted to identify the functional genes that have large effects on milk production traits to improve the accuracy of selection since the implementation of whole genomic selection. At the present, the RNA sequencing has been widely applied to study the specific gene expression patterns at different developmental stages or in different tissues [3]. We previously used this technique to obtain hepatic transcriptome of Chinese Holstein cows among dry period, early lactation, and peak of lactation, and found that the expression of the nucleobindin 2 (*NUCB2*) gene was significantly increased in early lactation (*q* value = 0.00811) and the peak of lactation (*q* value = 0.049246) as compared to dry period, respectively [4], so we considered that the *NUCB2* might be associated with milk production traits.

Nucleobindin 2 (NUCB2) is a Ca^2+^ binding protein, and it processes to generate a terminal peptide, termed nesfatin-1. Nesfatin-1 regulates the appetite and glucose metabolisms in humans and domestic animals [5,6,7]. *NUCB2* exhibits a high conservation among species, and its mRNA is ubiquitously expressed [8]. Sun et al. first demonstrated that *NUCB2* mRNA and nesfatin-1 protein were highly expressed in the liver of mouse fetus at embryonic day (E) 10.5, and their expression were extensively decreased at E17.5, which suggests that *NUCB2* might play a critical role in liver development and physiological functions in the developmental process of mouse fetus [9]. *NUCB2* is involved in human mammary epithelial cell proliferation and migration [10], and its expression is down-regulated when the milk production is decreased [11], which implies that the expression of *NUCB2* might be beneficial to the increase of milk production. In addition, the *NUCB2* gene was located on chr.15:43.5418 and it had a distance of 0.86 and 2.54 cM to the peak of reported quantitative trait locus (QTL) regions that have large effect on protein percentage [12] and protein yield [13], respectively. The *NUCB2* was also near to three significant SNPs for milk traits, BFGL-NGS-116109, BTB-00590405, and BTB-00590603, with the distance of 0.93–5.99 Mb [14]. These data indicate that *NUCB2* may be involved in milk production traits.

To date, rare studies have been reported to uncover the genetic association between the single nucleotide polymorphisms (SNPs) of *NUCB2* and milk production traits in dairy cattle. In addition, the studies have revealed that haplotype blocks that are formed by the SNPs have important implications for identifying associations with complex traits [15,16,17]. Hence, in this study, we detected the polymorphisms of the *NUCB2* gene and estimated the haplotype block that was formed by the identified SNPs, and then analyzed their associations with five milk traits, including milk yield, fat yield, fat percentage, protein yield, and protein percentage.

## 2. Materials and Methods

### 2.1. Animals

We selected a total of 1067 Chinese Holstein cows of 40 sire families from 22 dairy farms of the Sanyuan Lvhe Dairy Farming Centre (Beijing, China) on the same feeding conditions. The cows in each sire family were distributed in different dairy farms. These 1067 cows have three-generation pedigree information at least and Dairy Herd Improvement (DHI) records for 305-day milk yield, protein yield, protein percentage, fat yield, and fat percentage. Ethics Approval: The Institutional Animal Care and Use Committee (IACUC) of China Agricultural University approved all of the protocols for sample collection (permit number: DK996).

### 2.2. Phenotypic Data Collection

The Beijing Dairy Cattle Centre provided the phenotypic data of 305-day milk yield, fat yield, fat percentage, protein yield, and protein percentage in the first and second lactations (http://www.bdcc.com.cn/), and the descriptive statistics of them are shown in Table S1.

### 2.3. DNA Extraction

The semen DNAs of 40 sires were extracted using the salt-out procedures, and the blood DNAs of 1067 daughters were extracted while using the TIANamp Blood DNA Kit (Tiangen, Beijing, China), according to the manufacturer’s instructions. Subsequently, we measured the quantity and quality of the extracted DNAs by the NanoDrop 2000 spectrophotometer (Thermo Scientific, Hudson, NH, USA) and gel electrophoresis, respectively.

### 2.4. SNP Identification and Genotyping

We designed the primers (Table S2) to amplify the entire coding region and 2000 bp of the 5′ and 3′ flanking regions based on the bovine reference genome sequences of *NUCB2* by Primer3 (version 0.4.0, Whitehead Institute for Biomedical Research, Cambridge, MA, USA) (http://bioinfo.ut.ee/primer3-0.4.0/), and then synthesized them in the Beijing Genomics Institute (BGI, Beijing, China). We randomly mixed 40 semen DNAs into two pools (20 sires per pool) with equal concentrations (50 ng/µL) for each DNA. The reaction conditions and amplification procedures for PCR are provided in the Table S2. Afterwards, the PCR products were purified and sequenced by the ABI3730XL DNA analyser (Applied Biosystems, Foster City, CA, USA). Subsequently, we analyzed the sequencing data while using CHROMAS (version 2.23, Technelysium, Tewantin, Queensland, Australia) to discovery the potential SNPs. The identified SNPs were individually genotyped using Sequenom MassArray (Agena Bioscience, San Diego, CA, USA) for all the 1067 cows by matrix-assisted laser desorption/ionization time of flight mass spectrometry (MALDI-TOF MS, Bioyong Technologies Inc., HK).

### 2.5. Estimation of Linkage Disequilibrium (LD) and Association Analyses

The extent of LD between the identified SNPs was estimated while using Haploview 4.2 (Broad Institute of MIT and Harvard, Cambridge, MA, USA). Subsequently, we used the Statistical Analysis System (SAS) 9.13 software (SAS Institute, Cary, NC, USA) to analyze the associations of the SNPs and haplotype blocks with milk production traits with the following animal model: Y=μ+hys+b×M+G+a+e, in which, Y is the phenotypic value of each trait for each cow; μ is the overall mean; hys is the fixed effect of farm, year, and season; b is the regression coefficient of covariant M; M is the fixed effect of calving month; G is the genotype or haplotype combination effect; a is the individual random additive genetic effect, being distributed as $N\;\left(0,A\delta_{a}^{2}\right)$, with the additive genetic variance $\delta_{a}^{2}$; and, e is the random residual, which is distributed as $N\;\left(0,I\delta_{e}^{2}\right)$, with identity matrix I and residual error variance $\delta_{e}^{2}$. Bonferroni correction was performed for multiple testing, and the significant level was equal to the raw *P* value, divided by number of genotypes or haplotype combinations. In addition, the additive (a), dominant (d), and substitution (α) effects were calculated, as follows: $a=\frac{\mathrm{AA}-\mathrm{BB}}{2};\;d=\mathrm{AB}-\frac{\mathrm{AA}+\mathrm{BB}}{2};\;\alpha =a+d\;\left(q-p\right)$, where, AA, BB, and AB are the least square means of the milk production traits in the corresponding genotypes, p is the frequency of allele A, and q is the frequency of allele B.

### 2.6. Transcription Factor Binding Sites (TFBSs) Prediction

We used the MatInspector (version 3.11, Genomatix, Munich, Germany) (http://www.genomatix.de/cgi-bin/welcome/welcome.pl?s=d1b5c9a9015b02bb3b1a806f9c03293f) to predict the changes of the TFBSs that are caused by the identified SNPs in the 5′ flanking region of the *NUCB2* (matrix similarity threshold, MST > 0.85).

### 2.7. NUCB2 Gene Expression Analysis

Three healthy lactating Chinese Holstein cows were selected from the Sanyuanlvhe Dairy Farming Center (Beijing, China), and the heart, liver, spleen, lung, kidney, ovary, mammary, and uterus from each cow were collected. Subsequently, the expression of *NUCB2* in eight tissues was detected to further reveal its potential function. The total RNAs of the tissues were extracted while using a Trizol reagent (Invitrogen, Carlsbad, CA, USA), and the quantity and quality of the RNA were measured with a NanoDrop 2000 spectrophotometer (Thermo Scientific, Hudson, DE, USA) and gel electrophoresis, respectively. We used a PrimerScriptH RT reagent Kit (TaKaRa Biotechnology Co., Ltd., Dalian, China) for the reverse transcription. The qPCR primers of *NUCB2* and two reference genes (Ribosomal Protein S9, *RPS9*, and Ubiquitously Expressed Prefoldin Like Chaperone, *UXT*) were presented in Table S2. We conducted the qPCR while using a LightCycler^®^ 480 II (Roche, Penzberg, Germany) with the procedures that are shown in Table S2. All of the measurements were performed in triplicate and the relative gene expression was normalized by the *RPS9* and *UXT* with 2^-ΔΔCt^ method.

## 3. Results

### 3.1. SNP Identification in NUCB2 Gene

**Figure 1:**
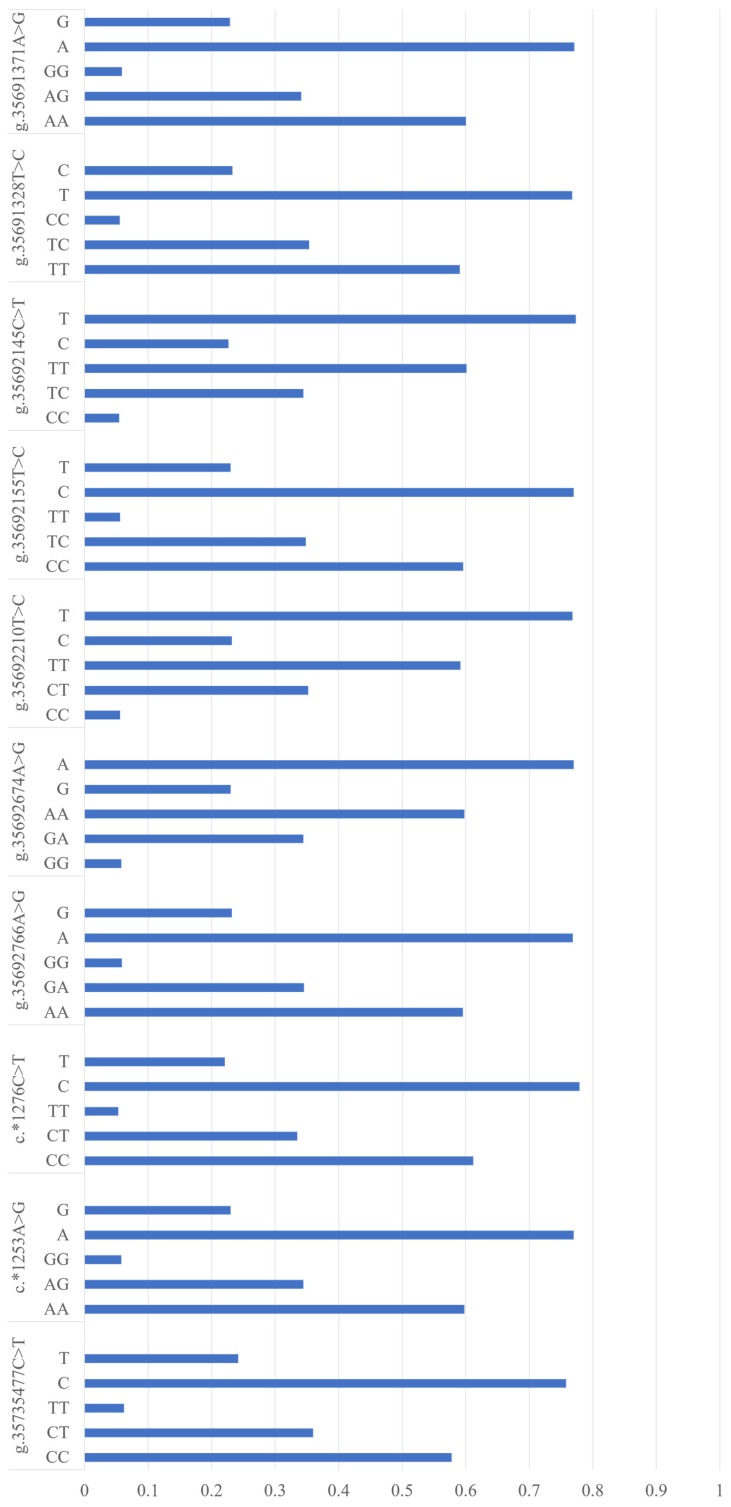
Allelic and genotypic frequencies of the ten single nucleotide polymorphisms (SNPs) in nucleobindin 2 (*NUCB2*). The “*” shows the SNPs located in the 3’untranslated region (UTR).

By re-sequencing, we identified ten SNPs in the entire coding region and 2000 bp of 5′ and 3′ flanking regions of *NUCB2*, including one in the 5′ flanking region, two in 3′ UTR, and seven in the 3′ flanking region (Table 1). Further, we individually genotyped these ten SNPs while using Sequenom MassArray for all the 1067 cows, and their allelic and genotypic frequencies are shown in Figure 1.

### 3.2. Associations between Ten SNPs and Five Milk Production Traits

We analyzed the genetic associations between the ten SNPs of *NUCB2* and milk yield, fat yield, fat percentage, protein yield, and protein percentage, respectively, by SAS, and the results showed that these ten SNPs were significantly associated with at least one milk traits in the first or second lactation (Table 2). In the first lactation, there were five SNPs that were associated with fat yield (*p* values: 0.0012–0.0178), nine with fat percentage (*p* values: 0.0035–0.0388), and eight with protein percentage (*p* values: 0.0075–0.0334), and no association was found between the SNPs and milk and protein yields (*p* values > 0.05). In the second lactation, ten SNPs had strong associations with milk yield (*p* values <= 1 × 10^−4^ and 0.0089), five with fat yield (*p* values: 0.0073–0.0466), one with fat percentage (*p* value = 0.0257), ten with protein yield (*p* values <= 1 × 10^−4^ and 0.0347), and six with protein percentage (*p* values: 0.0107–0.0467), respectively. Two SNPs, c.*1253A>G and g.35692674A>G, were strongly associated with fat yield (*p* values: 0.0073–0.0383) in both of the two lactations. The g.35735477C>T had significant association with fat percentage in the first (*p* value = 0.0122) and second lactation (*p* value = 0.0257). There were four SNPs, g.35735477C>T, c.*1276C>T, g.35692145C>T, and g.35691328T>C, which were associated with protein percentage ((*p* values: 0.0075–0.0357) in both lactations. In addition, as the results show in Table S3, the additive, dominant, and substitution effects of the ten SNPs were found to be significantly associated with milk traits (*p* values < 0.05).

### 3.3. Associations between Haplotype Combinations with Five Milk Traits

**Figure 2:**
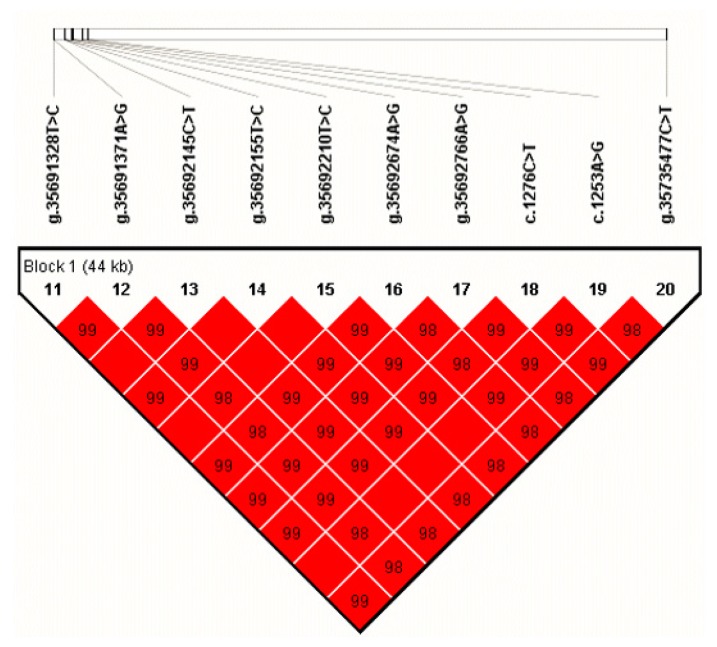
Linkage disequilibrium estimated among the SNPs of *NUCB2* (D′ = 0.98–1.00). The blocks indicate haplotype blocks and the text above the horizontal numbers is the SNP names. The values in boxes are pairwise SNP correlations (D′), while the boxes without numbers indicate complete linkage disequilibrium (LD) (D′ = 1).

We estimated the LD among the identified SNPs of *NUCB2* and found a haplotype block (D′ =0.98–1.00; Figure 2) that was included all ten SNPs. The block consisted of three haplotypes, H1 (TACTTAACAC), H2 (TACTTAACAT), and H3 (CGTCCGGTGT), with the frequency of 75.3%, 1.3%, and 22.6%, respectively. By haplotype-based association analyses, we found that the haplotype block was significantly associated with protein percentage (*p* value = 0.0393) in the first lactation, milk yield (*p* value = 0.0002), fat percentage (*p* value = 0.047), protein yield (*p* value = 0.0015), and protein percentage (*p* value = 0.0386) in the second lactation, respectively (Table 3).

### 3.4. TFBSs Changed by g.35735477C>T of NUCB2

**Figure 3:**
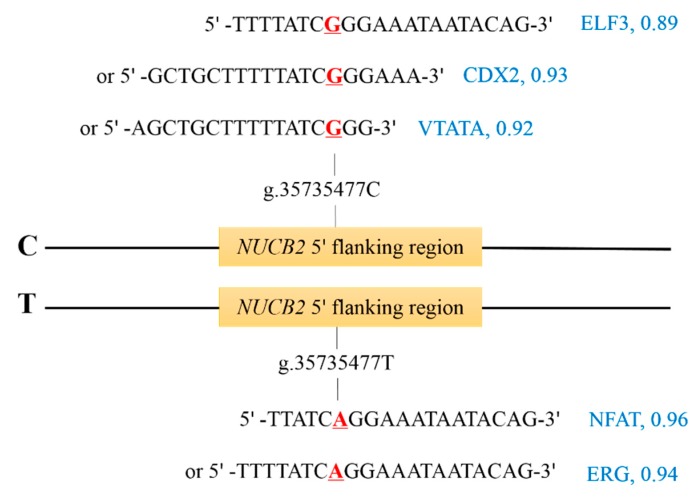
Transcription factor binding sites (TFBSs) in the 5′ flanking region of *NUCB2* gene. The nucleotides represent the transcription factor binding site sequences of ETS transcription factor 3 (ELF3), caudal type homeobox 2 (CDX2), mammalian C-type LTR TATA box (VTATA), nuclear factor of activated T-cells (NFAT), and v-ets erythroblastosis virus E26 oncogene homolog (ERG) with their matrix similarity over 0.85 (in blue), and the underlined nucleotide in red was the SNP.

For the SNP, g.35735477C>T, in the 5′ flanking region of the *NUCB2* gene, we predicted the changes of TFBSs caused by them while using Matinspector. The allele C in g.35735477C>T was predicted to create the binding sites for the transcription factors E74, like ETS transcription factor 3 (ELF3; MST = 0.89), caudal type homeobox 2 (CDX2; MST = 0.93), mammalian C-type LTR TATA box (VTATA; MST = 0.92), and the allele T in this SNP could create the TFBSs for nuclear factor of activated T-cells (NFAT; MST = 0.96) and v-ets erythroblastosis virus E26 oncogene homolog (ERG; MST = 0.94; Figure 3).

### 3.5. Expression of NUCB2 in Tissues

**Figure 4:**
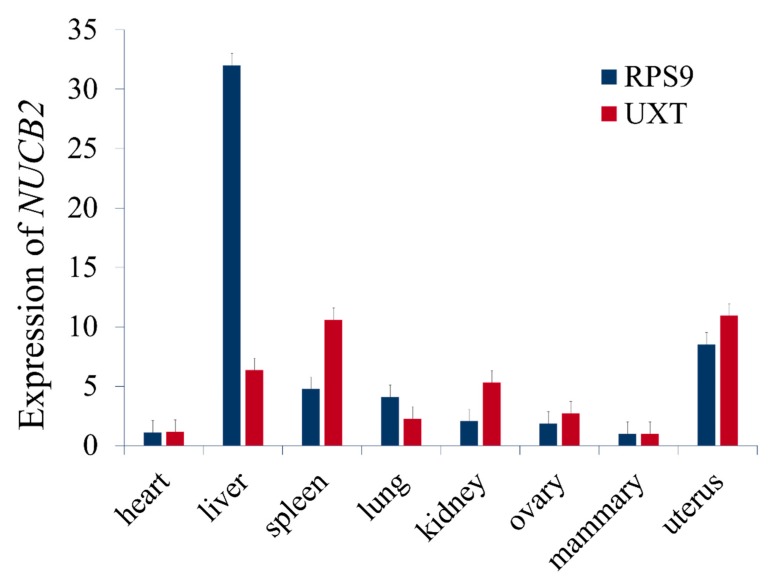
Expression of *NUCB2* gene in bovine tissues. Relative mRNA expression of *NUCB2* in eight tissues of lactating Holstein cows normalized by two reference genes, *RPS9* and *UXT*.

We found that *NUCB2* gene was expressed in heart, liver, spleen, lung, kidney, ovary, mammary gland, and uterus of the lactating Chinese Holstein and highly expressed in liver tissue while using the qPCR, (Figure 4), which suggested that it might be involved with substance metabolisms for milk synthesis in the liver.

## 4. Discussion

*NUCB2* was involved with milk production traits in dairy cows n our previous hepatic transcriptome study, and this follow-up investigation underlined that the SNPs of *NUCB2* have significant genetic effects on milk yield and composition.

Genomic selection has revolutionized dairy cattle breeding, and the evaluation system with DNA marker technology and genomics has doubled the rate of genetic progress for economic traits [18]. A study has revealed that the use of available loci improved the accuracy of genomic prediction in dairy cattle [19], which suggests that loci that are associated with milk production traits could be used in genomic selection programs that are aimed at accelerating genetic progress of dairy cattle. Studies have shown that the SNPs in the candidate genes significantly influenced the milk yield and composition in Holsteins [20,21,22,23,24,25]. In this study, we identified ten SNPs in *NUCB2* and found that the SNPs and haplotypes of this gene were significantly associated with milk production traits. Currently, the Illumina 50K/770K chip and the GeneSeek 90K/150K chip are commonly used, and most of the SNP markers in chips were from the SNP database with even distribution across the whole genome. Thus, the certain SNPs of *NUCB2* gene with significant genetic effects on milk traits could be put into the SNP chip to increase the selection efficiency for the milk production in dairy cattle.

In addition, for each identified SNP of *NUCB2* in the current study, we observed that the genotypic frequency of the cows with significantly high phenotypic values of 305-day milk yield, fat yield, fat percentage, protein yield, or protein percentage were higher. This implied the effectiveness of dairy cattle breeding in recent years, and also reflected that these loci might be closely associated with the milk production traits. However, we found that the ten SNPs of *NUCB2* showed different associations between the first and second lactations, and considered that the possible reason might be the different number of cows that are used for genetic association analysis, that is, we used 1067 cows in the first lactation and 740 in the second lactation (327 cows merely completed the milking of first lactation), which might impact the statistical significance. Generally, cows have higher milk production in the second lactation, and the different physiologic status of cows between the two lactations was also one possibility for the different genetic effects across lactations. In addition, the unstable association of the SNPs in two lactations was probably due to the fact that the causal polymorphisms are not very far, but not for these ones. Besides, we observed that the cows with heterozygous genotype showed the dominance for milk traits, ten SNPs for milk yield in the second lactation, for instance, which were caused by the dominance effects of the heterozygotes. We usually do not consider it in breeding, since the dominant effect is not heritable across generations. Hence, we should choose SNPs with significant additive effects on milk traits in all lactations for dairy cattle breeding.

In this study, we used the qPCR and observed that *NUCB2* was expressed in heart, liver, spleen, lung, kidney, ovary, mammary, and uterus tissues of dairy cows, and relatively highly expressed in liver. In dairy cows, the liver participates in carbohydrate, fat, protein, and other substance metabolisms, through blood circulation, it provides cholesterol, triglyceride, and other ingredients to mammary gland to synthesize milk protein and fat [26,27,28]. Our data suggest that *NUCB2* may play an important role in milk synthesis metabolism in dairy cows.

The specific binding of transcription factors to the regulatory regions of the DNAs is a important regulatory mechanism that affects gene expression [29,30]. In the current study, the allele C of g.35735477C>T was predicted to create the binding sites for ELF3, CDX2, and VTATA, and the allele T to invent the binding sites for NFAT and ERG. Oettgen et al. revealed that ELF3 directly drives *SPRR2A* mRNA expression through binding to the sites in its promoter during terminal differentiation of the epidermis [31]. The overexpression of ELF3 transactivates the promoter of *KRT12* in the differentiated mouse corneal epithelium [32]. ELF3 binds to the miR-141-3p promoter and it suppresses its expression, thereby promoting the epithelial-mesenchymal transition in human hepatocellular carcinoma [33]. ELF3 directly binds Estrogen Receptor α (ERα) and it represses its transcriptional activity in ERα positive breast cancer, showing a tumour suppressive role [34]. Zhu et al. reported that CDX2 directly transactivated the oncogene *CDH-17* and promoted hepatocellular carcinoma cell proliferation [35]. CDX2 transactivates the expression of GSK-3β and Axin2 to inhibit the cell proliferation and tumor formation in colon cancer [36]. CDX2 binds to the distinct target genes and it activates their expression in developing versus human and adult mouse intestinal cells [37]. Ross et al. identified a large number of potential TFBSs in the 5′ flanking sequences of *SRY* gene in human and bovine, including the TFBS for VTATA in the TATA-binding protein factors family with the matrix similarity score of 0.923 in bovine [38]. The NFAT family consists of five members, NFAT1–NFAT5, first found in T lymphocytes as an inducible nuclear factor that could bind to the IL-2 promoter and transactivate its expression [39]. NFAT proteins frequently cooperate with other transcriptional partners due to its weak DNA-binding capacity, and reports have shown the specific molecules that are up-/down-regulated by the different NFATs [40]. ERG regulates the expression of *SMAD2/3* to cause endothelial-dependent liver fibrogenesis [41], and it promotes endothelial homeostasis via the regulation of lineage-specific enhancers and super-enhancers [42]. These data indicate that these five transcription factors can regulate the expression of target genes by binding the TFBSs. Therefore, we speculated that the g.35735477C>T could affect the expression of *NUCB2* by the coactions of transcription factors ELF3, CDX2, VTATA, NFAT, and ERG. However, the further functional validation should be performed to verify the regulatory roles of *NUCB2* and its polymorphisms on milk traits in dairy cattle.

## 5. Conclusions

This is the first study to demonstrate the genetic associations of SNPs of *NUCB2* gene with milk production traits in dairy cows, and to show that g.35735477C>T might change the TFBSs of *NUCB2*. Our findings provide valuable molecular information for dairy cattle breeding, and the significant SNPs could be put into the SNP chip for genomic selection to improve the milk production in dairy cows.

## Figures and Tables

**Table 1:** Details about the identified SNPs of *NUCB2.*

SNP Name	GenBank No.	Location	Position (UMD3.1)	Mutation
g.35735477C>T	rs135882628	5′flanking region	chr15:35735477	C/T
c.*1253A>G	rs207689152	3′UTR	chr15:35693831	A/G
c.*1276C>T	rs208861167	3′UTR	chr15:35693399	C/T
g.35692766A>G	rs109930800	3′flanking region	chr15:35692766	A/G
g.35692674A>G	rs109930800	3′flanking region	chr15:35692674	A/G
g.35692210T>C	rs208420141	3′flanking region	chr15:35692210	T/C
g.35692155T>C	rs210564196	3′flanking region	chr15:35692155	T/C
g.35692145C>T	rs209421884	3′flanking region	chr15:35692145	C/T
g.35691328T>C	rs210746263	3′flanking region	chr15:35691328	T/C
g.35691371A>G	rs207595426	3′flanking region	chr15:35691371	A/G

Note: UTR: untranslated region. The “*” shows the SNPs located in the 3’ UTR.

**Table 2:** Associations of ten SNPs of *NUCB2* gene with milk production traits in two lactations in Chinese Holstein (LSM ± SE).

SNP	Lactation	Genotype (No.)	Milk Yield (kg)	Fat Yield (kg)	Fat Percentage (%)	Protein Yield (kg)	Protein Percentage (%)	No. of Significant SNPs
g.35735477C>T	1	CC (604)	10326 ± 63.16	343.09 ± 2.81	3.33 ± 0.03 ^Aa^	306 ± 2.05	2.96 ± 0.01 ^a^	2
		CT (376)	10309 ± 68.49	340.27 ± 3.02	3.31 ± 0.03 ^ab^	305.52 ± 2.2	2.96 ± 0.01 ^a^	
		TT (65)	10456 ± 111.13	338.57 ± 4.65	3.21 ± 0.05 ^Bb^	306.19 ± 3.39	2.92 ± 0.02 ^b^	
		*p* value	0.3565	0.285	0.0122	0.9428	0.0327	
	2	CC (415)	10617 ± 67.14 ^Aa^	385.18 ± 2.99	3.65 ± 0.03 ^a^	315.66 ± 2.18 ^b^	2.99 ± 0.01 ^Aa^	4
		CT (258)	10871 ± 72.95 ^B^	387.94 ± 3.21	3.59 ± 0.03 ^b^	320.54 ± 2.33 ^a^	2.96 ± 0.01 ^Bb^	
		TT (49)	10475 ± 126.69 ^Aa^	383.05 ± 5.25	3.67 ± 0.05 ^ab^	309.98 ± 3.83^b^	2.97 ± 0.02 ^ab^	
		*p* value	<0.0001	0.4542	0.0257	0.0036	0.0107	
c.*1253A>G	1	AA (622)	10377 ± 62.96	346.91 ± 2.81 ^a^	3.35 ± 0.03 ^a^	308.01 ± 2.05	2.96 ± 0.01 ^a^	3
		AG (358)	10330 ± 67.85	343.52 ± 2.99 ^ab^	3.33 ± 0.03 ^ab^	306.64 ± 2.17	2.96 ± 0.01 ^a^	
		GG (60)	10411 ± 113.36	335.92 ± 4.73 ^b^	3.23 ± 0.05 ^b^	304.51 ± 3.44	2.92 ± 0.02 ^b^	
		*p* value	0.5823	0.0178	0.0167	0.4162	0.0334	
	2	AA (434)	10739 ± 65.59 ^Aa^	389.07 ± 3.02 ^a^	3.63 ± 0.03	319.03 ± 2.12 ^a^	2.98 ± 0.01	3
		AG (248)	10934 ± 73.57 ^B^	396.99 ± 3.26 ^b^	3.6 ± 0.03	322.97 ± 2.34 ^Aa^	2.96 ± 0.01	
		GG (45)	10468 ± 130.21 ^Aa^	386.53 ± 5.54 ^ab^	3.66 ± 0.05	308.67 ± 3.93 ^Bb^	2.96 ± 0.02	
		*p* value	0.0002	0.0073	0.3202	0.0006	0.089	
c.*1276C>T	1	CC (632)	10332 ± 62.27	346.1 ± 2.78	3.36 ± 0.03 ^a^	305.38 ± 2.03	2.96 ± 0.01 ^a^	3
		CT (346)	10320 ± 68.73	343.57 ± 3.02	3.34 ± 0.03 ^ab^	305.67 ± 2.2	2.96 ± 0.01 ^a^	
		TT (55)	10392 ± 113.98	336.79 ± 4.74	3.25 ± 0.05 ^b^	302.85 ± 3.46	2.92 ± 0.02 ^b^	
		*p* value	0.7939	0.0661	0.0278	0.6665	0.0217	
	2	CC (442)	10687 ± 65.62 ^a^	386.11 ± 2.93	3.63 ± 0.03	316.61 ± 2.13 ^ab^	2.98 ± 0.01 ^a^	3
		CT (238)	10850 ± 74 ^b^	388.08 ± 3.23	3.59 ± 0.03	319.51 ± 2.36 ^a^	2.96 ± 0.01 ^b^	
		TT (42)	10521 ± 130.66 ^a^	384.93±5.4	3.67 ± 0.05	309.97 ± 3.94 ^b^	2.95 ± 0.02 ^ab^	
		*p* value	0.0089	0.7061	0.1478	0.0347	0.0357	
g.35692766A>G	1	AA (626)	10418 ± 63.52	347.12±2.84	3.35 ± 0.03^a^	307.67 ± 2.07	2.96 ± 0.01	1
		GA (363)	10345 ± 68.1	343.28±3	3.33 ± 0.03^ab^	305.79 ± 2.19	2.96 ± 0.01	
		GG (62)	10510 ± 112.05	339.43±4.68	3.24 ± 0.05^b^	306.98 ± 3.41	2.92 ± 0.02	
		*p* value	0.1772	0.0623	0.0382	0.4738	0.0638	
	2	AA (434)	10713 ± 65.44 ^Aa^	386.64±2.91	3.63 ± 0.03	318.28 ± 2.12 ^b^	2.98 ± 0.01 ^a^	4
		GA (249)	10930 ± 72.31 ^B^	392.91±3.16	3.61 ± 0.03	323.49 ± 2.3 ^Aa^	2.96 ± 0.01 ^ab^	
		GG (46)	10498 ± 130.83 ^Aa^	381.61±5.43	3.66 ± 0.05	308.07 ± 3.96 ^Ba^	2.94 ± 0.02 ^b^	
		*p* value	0.0002	0.018	0.4651	<0.0001	0.0467	
g.35692674A>G	1	CC (60)	10345 ± 113.56	332.97±4.74^Aa^	3.23 ± 0.05^a^	301.01 ± 3.45	2.92 ± 0.02 ^a^	3
		CT (358)	10330 ± 67.9	343.02±2.99^ab^	3.33 ± 0.03^ab^	305.25 ± 2.18	2.96 ± 0.01 ^b^	
		TT (622)	10348 ± 62.26	345.78±2.78^Bb^	3.36 ± 0.03^b^	305.7 ± 2.02	2.96 ± 0.01 ^b^	
		*p* value	0.9433	0.0081	0.0116	0.3135	0.0273	
	2	CC (45)	10599 ± 129.91 ^b^	389.01±5.38^ab^	3.68 ± 0.05	313.59 ± 3.92 ^Aa^	2.96 ± 0.02	3
		CT (248)	10973 ± 73.35 ^Aa^	395.5±3.21^a^	3.61 ± 0.03	324.92 ± 2.34 ^B^	2.96 ± 0.01	
		TT (434)	10733 ± 65.85 ^Bb^	388.86±2.93^b^	3.63 ± 0.03	318.92 ± 2.13 ^Aa^	2.98 ± 0.01	
		*p* value	0.0002	0.0383	0.3137	0.0009	0.1688	
g.35692210T>C	1	CC (59)	10441 ± 113.16	337.01 ± 4.71	3.24 ± 0.05	305.33 ± 3.43	2.93 ± 0.02	0
		CT (372)	10325 ± 68.18	340.45 ± 3.01	3.31 ± 0.03	305.34 ± 2.19	2.96 ± 0.01	
		TT (625)	10373 ± 63.73	344.35 ± 2.85	3.34 ± 0.03	306.62 ± 2.08	2.96 ± 0.01	
		*p* value	0.4509	0.0683	0.0533	0.6822	0.0824	
	2	CC (44)	10443 ± 132.87 ^Aa^	377.54 ± 5.51 ^a^	3.64 ± 0.05	307.55 ± 4.02 ^Aa^	2.95 ± 0.02 ^ab^	4
		CT (254)	10916 ± 73.07 ^B^	388.6 ± 3.21 ^b^	3.58 ± 0.03	321.67 ± 2.33 ^Bb^	2.96 ± 0.01 ^a^	
		TT (434)	10688 ± 66.14 ^Aa^	383.07 ± 2.95 ^a^	3.61 ± 0.03	316.51 ± 2.15 ^a^	2.98 ± 0.01^b^	
		*p* value	<0.0001	0.0331	0.3355	0.0003	0.0143	
g.35692155T>C	1	CC (620)	10297 ± 63.03	344.52 ± 2.83 ^Aa^	3.35 ± 0.03 ^a^	304.35 ± 2.05	2.96 ± 0.01 ^a^	3
		TC (362)	10284 ± 67.63	341.51 ± 3 ^a^	3.32 ± 0.03 ^ab^	304.36 ± 2.17	2.96 ± 0.01 ^a^	
		TT (58)	10378 ± 114.2	328.5 ± 4.92 ^Bb^	3.24 ± 0.05 ^b^	302.2 ± 3.46	2.92 ± 0.02 ^B^	
		*p* value	0.6782	0.0012	0.0144	0.7741	0.0138	
	2	CC (432)	10690 ± 65.94 ^Aa^	386.06 ± 2.94	3.63 ± 0.03	317.22 ± 2.14 ^ab^	2.98 ± 0.01	2
		TC (249)	10889 ± 73.19 ^B^	390.76 ± 3.2	3.6 ± 0.03	321.46 ± 2.33 ^Aa^	2.96 ± 0.01	
		TT (44)	10474 ± 131.19 ^Aa^	382.27 ± 5.43	3.66 ± 0.05	308.69 ± 3.96 ^Bb^	2.95 ± 0.02	
		*p* value	0.0006	0.109	0.3315	0.0018	0.0989	
g.35692145C>T	1	CC (57)	10447 ± 113.95	338.63 ± 4.74	3.26 ± 0.05 ^a^	303.9 ± 3.45	2.91 ± 0.02 ^a^	2
		TC (359)	10342 ± 68.09	343.23 ± 3	3.33 ± 0.03 ^ab^	305.98 ± 2.18	2.96 ± 0.01 ^b^	
		TT (627)	10366 ± 63.35	346.42 ± 2.83	3.36 ± 0.03 ^b^	306.41 ± 2.06	2.96 ± 0.01 ^b^	
		*p* value	0.6061	0.0937	0.0388	0.7116	0.0133	
	2	CC (43)	10461 ± 131.5 ^Aa^	383.01 ± 5.44^ab^	3.67 ± 0.05	309.35 ± 3.97 ^Aa^	2.95 ± 0.02 ^ab^	3
		TC (247)	10948 ± 72.69 ^B^	392.58 ± 3.18^a^	3.59 ± 0.03	323.82 ± 2.31 ^Bb^	2.96 ± 0.01 ^a^	
		TT (439)	10692 ± 65.5 ^Aa^	386.85 ± 2.92^b^	3.64 ± 0.03	318.16 ± 2.13 ^a^	2.99 ± 0.01 ^b^	
		*p* value	<0.0001	0.0466	0.1716	0.0002	0.0185	
g.35691328T>C	1	AA (626)	10310 ± 63.52	347.08 ± 2.81^Aa^	3.36 ± 0.03 ^a^	304.24 ± 2.07	2.96 ± 0.01 ^a^	3
		AG (375)	10289 ± 68.03	344.2 ± 2.97^a^	3.33 ± 0.03 ^ab^	303.88 ± 2.18	2.96 ± 0.01 ^a^	
		GG (59)	10389 ± 112.86	331.3 ± 4.95^Bb^	3.25 ± 0.05 ^b^	302.57 ± 3.42	2.92 ± 0.02 ^b^	
		*p* value	0.6345	0.0014	0.0248	0.8566	0.0302	
	2	AA (436)	10631 ± 65.52 ^Aa^	384.07 ± 2.92	3.64 ± 0.03	316.66 ± 2.12 ^Aa^	2.99 ± 0.01 ^a^	3
		AG (254)	10910 ± 72.91 ^B^	388.98 ± 3.2	3.58 ± 0.03	322.6 ± 2.33 ^B^	2.96 ± 0.01 ^b^	
		GG (44)	10395 ± 131.93 ^Aa^	378.08 ± 5.47	3.66 ± 0.05	307.3 ± 3.99 ^Ab^	2.96 ± 0.02 ^ab^	
		*p* value	<.0001	0.0505	0.0698	<.0001	0.0175	
g.35691371A>G	1	AA (604)	10378 ± 62.51	345.22 ± 2.79 ^Aa^	3.34 ± 0.03 ^Aa^	306.3 ± 2.03	2.95 ± 0.01 ^Aa^	3
		AG (343)	10336 ± 68.31	341.23 ± 3.01 ^ab^	3.31 ± 0.03 ^a^	305.12 ± 2.19	2.95 ± 0.01 ^a^	
		GG (59)	10417 ± 112.77	332.6 ± 4.7 ^Bb^	3.2 ± 0.05B ^b^	302.27 ± 3.43	2.91 ± 0.02 ^Bb^	
		*p* value	0.6244	0.0048	0.0035	0.3689	0.0075	
	2	AA (435)	10755 ± 66.43 ^b^	388.73 ± 2.96	3.63 ± 0.03	320.29 ± 2.16 ^ab^	2.98 ± 0.01	2
		AG (246)	10938 ± 73.48 ^Aa^	391.8 ± 3.22	3.59 ± 0.03	324.21 ± 2.34 ^Aa^	2.96 ± 0.01	
		GG (45)	10506 ± 130.52 ^Bb^	385.76 ± 5.41	3.68 ± 0.05	311.74 ± 3.94^Bb^	2.96 ± 0.02	
		*p* value	0.0007	0.359	0.1113	0.0026	0.1356	
No. of significant SNPs	1		0	5	9	0	8	
	2		10	5	1	10	6	

Note: The number in the bracket represents the number of cows for the corresponding genotype; *p* value shows the significance for the genetic effects of SNPs; ^a, b^ within the same column with different superscripts means *p* value < 0.05; ^A, B^ within the same column with different superscripts means *p* value < 0.01.

**Table 3:** Associations of haplotype blocks with milk production traits in two lactations in Chinese Holstein (LSM ± SE).

Lactation	Haplotype Combination (No.)	Milk Yield (kg)	Fat Yield (kg)	Fat Percentage (%)	Protein Yield (kg)	Protein Percentage (%)
1	H1H1(603)	10420 ± 65.07	347.91 ± 2.9	3.35 ± 0.03	309.3 ± 2.11	2.96 ± 0.01 ^a^
	H1H3(350)	10363 ± 70.17	343.55 ± 3.09	3.33 ± 0.03	307.37 ± 2.25	2.96 ± 0.01 ^a^
	H3H3(59)	10495 ± 114.84	341.13 ± 4.79	3.26 ± 0.05	307.19 ± 3.49	2.93 ± 0.02 ^b^
	*p* value	0.358	0.066	0.0819	0.4332	0.0393
2	H1H1(416)	10654 ± 67.2 ^A^	384.36 ± 3	3.63 ± 0.03^a^	316.81 ± 2.18 ^Aa^	2.98 ± 0.01 ^a^
	H1H3(238)	10875 ± 74.21 ^B^	386.56 ± 3.24	3.57 ± 0.03^b^	321.19 ± 2.36 ^ab^	2.96 ± 0.01 ^b^
	H3H3(44)	10416 ± 133.65 ^A^	379.5 ± 5.54	3.66 ± 0.05^ab^	307.95 ± 4.04 ^Bb^	2.96 ± 0.02 ^ab^
	*p* value	0.0002	0.3786	0.047	0.0015	0.0386

Note: H means haplotype; the number in the bracket represents the number of cows for the corresponding haplotype combination; H1 (TACTTAACAC), H2 (TACTTAACAT), H3 (CGTCCGGTGT); *p* value shows the significance for genetic effects among the haplotype blocks; ^a, b^ within the same column with different superscripts means *p* value < 0.05; ^A, B^ within the same column with different superscripts means *p* value < 0.01.

## Supplementary Materials

The following are available online at https://www.mdpi.com/2073-4425/10/6/449/s1, Table S1: Descriptive statistics of the phenotypic values for milk production traits. Table S2: Primers and procedures for PCR and qPCR used in SNP identification. Table S3: Additive, dominant and allele substitution effects of SNPs on milk production traits in Chinese Holstein.

## References

[1] Tunick M.H., Van Hekken D.L. (2015). Dairy Products and Health: Recent Insights. J. Agric. Food Chem..

[2] Spelman R.J., Coppieters W., Karim L., van Arendonk J.A., Bovenhuis H. (1996). Quantitative trait loci analysis for five milk production traits on chromosome six in the Dutch Holstein-Friesian population. Genetics.

[3] Denoeud F., Aury J.M., Da Silva C., Noel B., Rogier O., Delledonne M., Morgante M., Valle G., Wincker P., Scarpelli C. (2008). Annotating genomes with massive-scale RNA sequencing. Genome Biol..

[4] Liang R., Han B., Li Q., Yuan Y., Li J., Sun D. (2017). Using RNA sequencing to identify putative competing endogenous RNAs (ceRNAs) potentially regulating fat metabolism in bovine liver. Sci. Rep..

[5] Oh S., Shimizu H., Satoh T., Okada S., Adachi S., Inoue K., Eguchi H., Yamamoto M., Imaki T., Hashimoto K. (2006). Identification of nesfatin-1 as a satiety molecule in the hypothalamus. Nature.

[6] Stengel A., Tache Y. (2011). Minireview: Nesfatin-1-An Emerging New Player in the Brain-Gut, Endocrine, and Metabolic Axis. Endocrinology.

[7] Ramesh N., Gawli K., Pasupulleti V.K., Unniappan S. (2017). Metabolic and Cardiovascular Actions of Nesfatin-1: Implications in Health and Disease. Curr. Pharm. Des..

[8] Mohan H., Unniappan S. (2013). Phylogenetic aspects of nucleobindin-2/nesfatin-1. Curr. Pharm. Des..

[9] Sun S., Yang H. (2018). Tissue-Specific Localization NUCB2/nesfatin-1 in the Liver and Heart of Mouse Fetus. Dev. Reprod..

[10] Suzuki S., Takagi K., Miki Y., Onodera Y., Akahira J., Ebata A., Ishida T., Watanabe M., Sasano H., Suzuki T. (2012). Nucleobindin 2 in human breast carcinoma as a potent prognostic factor. Cancer Sci..

[11] Boutinaud M., Galio L., Lollivier V., Finot L., Wiart S., Esquerre D., Devinoy E. (2013). Unilateral once daily milking locally induces differential gene expression in both mammary tissue and milk epithelial cells revealing mammary remodeling. Physiol. Genom..

[12] Schopen G.C., Koks P.D., van Arendonk J.A., Bovenhuis H., Visker M.H. (2009). Whole genome scan to detect quantitative trait loci for bovine milk protein composition. Anim. Genet..

[13] Daetwyler H.D., Schenkel F.S., Sargolzaei M., Robinson J.A.B. (2008). A genome scan to detect quantitative trait loci for economically important traits in Holstein cattle using two methods and a dense single nucleotide polymorphism map. J. Dairy Sci..

[14] Cole J.B., Wiggans G.R., Ma L., Sonstegard T.S., Lawlor T.J., Crooker B.A., Van Tassell C.P., Yang J., Wang S., Matukumalli L.K. (2011). Genome-wide association analysis of thirty one production, health, reproduction and body conformation traits in contemporary U.S. Holstein cows. BMC Genom..

[15] Hagenblad J., Tang C., Molitor J., Werner J., Zhao K., Zheng H., Marjoram P., Weigel D., Nordborg M. (2004). Haplotype structure and phenotypic associations in the chromosomal regions surrounding two Arabidopsis thaliana flowering time loci. Genetics.

[16] Nothnagel M., Rohde K. (2005). The effect of single-nucleotide polymorphism marker selection on patterns of haplotype blocks and haplotype frequency estimates. Am. J. Hum. Genet..

[17] Patil N., Berno A.J., Hinds D.A., Barrett W.A., Doshi J.M., Hacker C.R., Kautzer C.R., Lee D.H., Marjoribanks C., McDonough D.P. (2001). Blocks of limited haplotype diversity revealed by high-resolution scanning of human chromosome 21. Science.

[18] Wiggans G.R., Cole J.B., Hubbard S.M., Sonstegard T.S. (2017). Genomic Selection in Dairy Cattle: The USDA Experience. Annu. Rev. Anim. Biosci..

[19] Zhang Z., Ober U., Erbe M., Zhang H., Gao N., He J., Li J., Simianer H. (2014). Improving the accuracy of whole genome prediction for complex traits using the results of genome wide association studies. PLoS ONE.

[20] Bouwman A.C., Bovenhuis H., Visker M.H., van Arendonk J.A. (2011). Genome-wide association of milk fatty acids in Dutch dairy cattle. BMC Genet..

[21] Matsumoto H., Sasaki K., Bessho T., Kobayashi E., Abe T., Sasazaki S., Oyama K., Mannen H. (2012). The SNPs in the ACACA gene are effective on fatty acid composition in Holstein milk. Mol. Biol. Rep..

[22] Bhattarai D., Chen X., Ur Rehman Z., Hao X., Ullah F., Dad R., Talpur H.S., Kadariya I., Cui L., Fan M. (2017). Association of MAP4K4 gene single nucleotide polymorphism with mastitis and milk traits in Chinese Holstein cattle. J. Dairy. Res..

[23] Han B., Liang W., Liu L., Li Y., Sun D. (2017). Determination of genetic effects of ATF3 and CDKN1A genes on milk yield and compositions in Chinese Holstein population. BMC Genet..

[24] Han B., Liang W., Liu L., Li Y., Sun D. (2018). Genetic association of the ACACB gene with milk yield and composition traits in dairy cattle. Anim. Genet..

[25] Han B., Yuan Y., Liang R., Li Y., Liu L., Sun D. (2019). Genetic Effects of LPIN1 Polymorphisms on Milk Production Traits in Dairy Cattle. Genes (Basel).

[26] Bell A.W. (1995). Regulation of organic nutrient metabolism during transition from late pregnancy to early lactation. J. Anim. Sci..

[27] Rawson P., Stockum C., Peng L.F., Manivannan B., Lehnert K., Ward H.E., Berry S.D., Davis S.R., Snell R.G., McLauchlan D. (2012). Metabolic proteomics of the liver and mammary gland during lactation. J. Proteom..

[28] Moyes K.M., Bendixen E., Codrea M.C., Ingvartsen K.L. (2013). Identification of hepatic biomarkers for physiological imbalance of dairy cows in early and mid lactation using proteomic technology. J. Dairy Sci..

[29] Tugrul M., Paixao T., Barton N.H., Tkacik G. (2015). Dynamics of Transcription Factor Binding Site Evolution. PLoS Genet..

[30] Talebzadeh M., Zare-Mirakabad F. (2014). Transcription factor binding sites prediction based on modified nucleosomes. PLoS ONE.

[31] Oettgen P., Alani R.M., Barcinski M.A., Brown L., Akbarali Y., Boltax J., Kunsch C., Munger K., Libermann T.A. (1997). Isolation and characterization of a novel epithelium-specific transcription factor, ESE-1, a member of the ETS family. Mol. Cell Biol..

[32] Yoshida N., Yoshida S., Araie M., Handa H., Nabeshima Y. (2000). Ets family transcription factor ESE-1 is expressed in corneal epithelial cells and is involved in their differentiation. Mech. Deve..

[33] Zheng L., Xu M., Xu J., Wu K., Fang Q., Liang Y., Zhou S., Cen D., Ji L., Han W. (2018). ELF3 promotes epithelial-mesenchymal transition by protecting ZEB1 from miR-141-3p-mediated silencing in hepatocellular carcinoma. Cell Death Dis..

[34] Gajulapalli V.N.R., Samanthapudi V.S.K., Pulaganti M., Khumukcham S.S., Malisetty V.L., Guruprasad L., Chitta S.K., Manavathi B. (2016). A transcriptional repressive role for epithelial-specific ETS factor ELF3 on oestrogen receptor α in breast cancer cells. Biochem. J..

[35] Zhu R., Wong K.F., Lee N.P., Lee K.F., Luk J.M. (2010). HNF1alpha and CDX2 transcriptional factors bind to cadherin-17 (CDH17) gene promoter and modulate its expression in hepatocellular carcinoma. J. Cell Biochem..

[36] Yu J.H., Liu D., Sun X.J., Yang K., Yao J.F., Cheng C., Wang C.B., Zheng J.B. (2019). CDX2 inhibits the proliferation and tumor formation of colon cancer cells by suppressing Wnt/β-catenin signaling via transactivation of GSK-3 β and Axin2 expression. Cell Death Dis..

[37] Kumar N., Tsai Y.H., Chen L., Zhou A., Banerjee K.K., Saxena M., Huang S., Toke N.H., Xing J., Shivdasani R.A. (2019). The lineage-specific transcription factor CDX2 navigates dynamic chromatin to control distinct stages of intestine development. Development.

[38] Ross D.G.F., Bowles J., Koopman P., Lehnert S. (2008). New insights into SRY regulation through identification of 5 conserved sequences. BMC Mol. Biol..

[39] Shaw J.P., Utz P.J., Durand D.B., Toole J.J., Emmel E.A., Crabtree G.R. (1988). Identification of a putative regulator of early T cell activation genes. Science.

[40] Mognol G.P., Carneiro F.R., Robbs B.K., Faget D.V., Viola J.P. (2016). Cell cycle and apoptosis regulation by NFAT transcription factors: New roles for an old player. Cell Death Dis..

[41] Dufton N.P., Peghaire C.R., Osuna-Almagro L., Raimondi C., Kalna V., Chuahan A., Webb G., Yang Y.W., Birdsey G.M., Lalor P. (2017). Dynamic regulation of canonical TGF β signalling by endothelial transcription factor ERG protects from liver fibrogenesis. Nat. Commun..

[42] Kalna V., Yang Y., Peghaire C., Frudd K., Hannah R., Shah A.V., Osuna Almagro L., Boyle J.J., Gottgens B., Ferrer J. (2019). The Transcription Factor ERG Regulates Super-Enhancers Associated with an Endothelial-Specific Gene Expression Program. Circ. Res..

